# Dynamic in vitro measurement of patellar movement after total knee arthroplasty: an in vitro study

**DOI:** 10.1186/1471-2474-6-30

**Published:** 2005-06-15

**Authors:** Sven Ostermeier, Olaf Buhrmester, Christof Hurschler, Christina Stukenborg-Colsman

**Affiliations:** 1 Department of Orthopaedics Hannover Medical School (MHH) Hannover, Germany

## Abstract

**Background:**

Changing the kinematic behaviour of patellar movement could be one of the reasons for anterior knee pain after implantation of a total knee arthroplasty (TKA). The aim of the current study was to measure the potential influence on patellar kinematics of patellar resurfacing during TKA.

**Methods:**

Patellar movement before and after TKA with and without patellar resurfacing was measured under dynamic conditions in an *in vitro *cadaver simulation. Physiologic Musculus quadriceps forces were applied to five physiologic human knee specimens undergoing simulated isokinetic extension motions, patellar movement was measured using an ultrasonic measurement system. Thereafter, the Interax^® ^I.S.A.-prosthesis system was implanted without and with resurfacing the patella, and patellar movement was again measured.

**Results:**

The physiologic patella center moved on a semilunar path up to 6.4 mm (SD 6.4 mm) medially during extension. After TKA, the unresurfaced patella showed significantly less medial translation (p = 0.04) than the resurfaced patella. Subsequent resurfacing of the patella then resulted in a return to mediolateral positioning of the patella similar to the physiological case, whereas the resurfaced patella tilted up to twice as much as physiologic.

**Conclusion:**

The results of this study suggest that resurfacing of the patella during TKA can result in a restoration of the physiologic mediolateral shift of the patellofemoral joint while angulation of the patella remains unphysiologic.

## Background

Patellofemoral or anterior knee pain represents one of the most common problems during rehabilitation after total knee arthroplasty (TKA), with postoperative problems reported in 0,5–12% of patients [1-6]. Although the components of the prosthesis may have been implanted correctly, with postoperative radiographs showing no malalignment, patients were often unable to flex the knee or bear weight on the treated joint [5,6]. While many authors observed a theoretical advantage of primary resurfacing of the backside of the patella during TKA in avoiding these problems [1-4,7], others disagreed, and do not recommend routine resurfacing of the patella [8-10]. Furthermore, some authors suggest that only through a correctly implanted patella inlay it is possible to avoid changing patellar tracking in the femoral groove and with it the associated potential malalignment of the degenerated patella [1,11-13].

Several types of retropatellar resurfacing devices have been developed. These included symmetrical, dome shaped, and non anatomic all polyethylene components [3]. Other devices had a metal-backed baseplate with a rotating, asymmetrical high-conforming polyethylene prosthesis [14]. In-vitro studies utilizing pressure-sensitive films showed significantly higher stresses in these designs which exceeded the yield strength on the polyethylene (>12 MPa) [9,14-17]. Furthermore, other studies showed substantial changes in movement of the patella after TKA: An unresurfaced patella moved along a different path in the groove of the femoral component compared to the physiological motion [1]. The patella shifted less medially than the physiologic patella in the physiologic femoral groove, although the angulation of the patella was not changed [1]. Thus, while demonstrating the same amount of flexion around the horizontal axis and rotation around the sagittal axis, the resurfaced patella moved on the same path as a physiologic patella, while at the same time exhibiting a significantly greater lateral tilt around the frontal axis [11-13].

Nonetheless, none of these studies compared the kinematics of the physiologic patella to those of an (un)resurfaced patella after TKA under physiologic loads and dynamic conditions. This study was therefore conducted to measure the path and rotational movement of a physiologic patella, and to compare that motion to the unresurfaced and resurfaced patella after TKA.

## Methods

**Figure 1:**
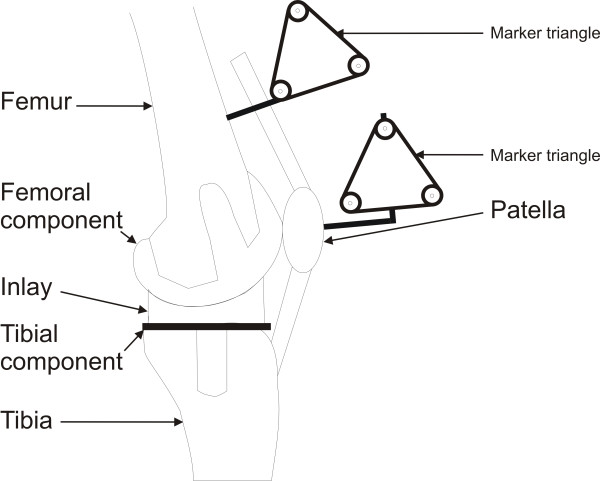
Implanted components with attached marker triangles

**Figure 2:**
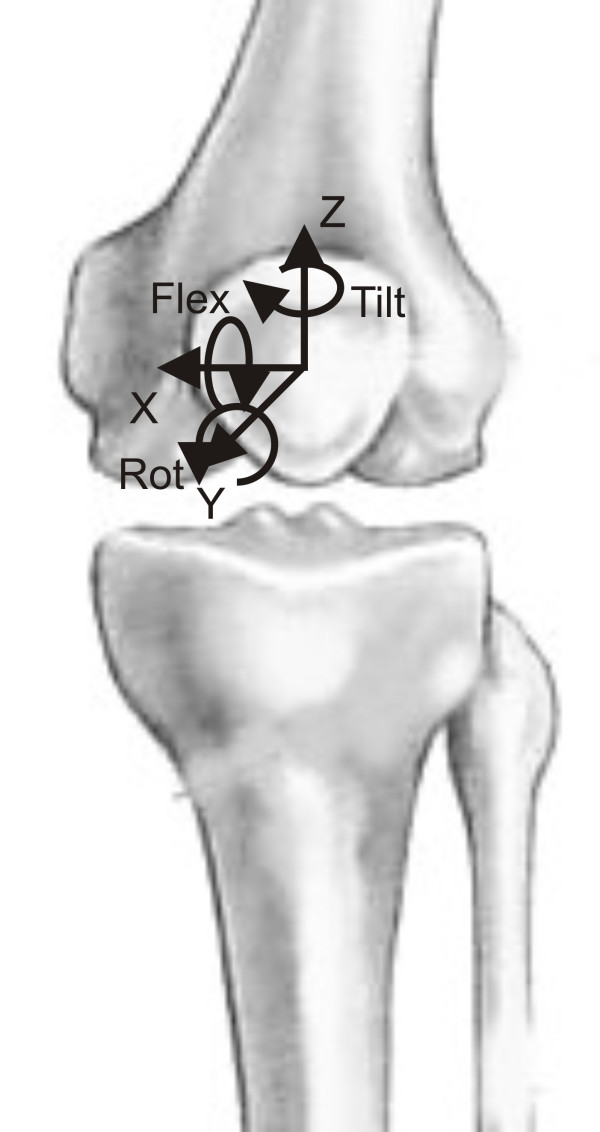
Directions of translation and rotation of the patellar center

Five (5) fresh frozen left knee specimens (mean age 62, range 52–75 years) were mounted into a specially designed knee simulator in which isokinetic flexion-extension motions were simulated as described by Stukenborg-Colsman *et al. *before [18]. As this test setup performed an *in vitro *test no ethic approval was necessary. Each knee specimen was transected approximately 30 cm distal and proximal to the knee joint line. The skin and subcutaneous tissues were removed, preserving the muscles, articular capsule, ligaments, and tendons. Movement of the patella in relation to the femur was measured using an ultrasound based three-dimensional motion analysis system (Zebris CMS-100, Isny, Germany). Marker triangles with a leg length of 50 mm were attached to the femur and the patella by means of uni-cortical screws (Fig. 1). Each marker triangle consisted of three ultrasound emitters placed on the edges of each triangle, and marker position was dynamically measured at a sampling frequency of 10 Hz by an H-shaped array of microphones placed at a distance of 1500 mm medial to the specimen. With this setup, the ultrasound positioning system is reported by the manufacturer to produce a theoretical accuracy of 0.1 mm for translation and 0.1 degrees for rotation of the triangles. The motions of each triangle were geometrically transformed to the geometrical center of the bony patella and the center of the femoral epicondylar axis. Thus, the relative movement of the center of the patella with respect to the center of the femur was measured with directions defined as follows: mediolateral [x], proximodistal [y] and anteroposterior [z] translation. An internal rotation along the long axis of the patella was defined as tilt, a forward rotation along the horizontal axis as flexion and an external rotation along the sagittal axis as rotation (Fig. 2).

The knee specimens were oriented with the femur fixed horizontally and the patella facing downwards. The tibia was attached to the simulator at mid-length by means of a linear-rotational bearing which permits axial sliding and turning as well as rotation transverse to the axis of the tibia. The bearing in turn was attached to a swing arm which allows varus-valgus rotation. The swing arm was equipped with a strain-gage based load measuring device which allows the extending moment applied to the tibia to be monitored continuously as described by Stukenborg-Colsman *et al. *[18].

Movement of the tibia was generated by the coordinated activation of two hydraulic cylinders, one to simulate quadriceps muscle force, and the other to apply external flexion moment. The quadriceps force was transmitted through a special clamp which was attached on the quadriceps tendon. An isokinetic extension cycle is simulated from 120 degrees flexion to full extension of the knee with an extension moment of 31 Nm [18]. During this extension cycle the cylinder which simulated the quadriceps force, was required to generated forces of up to 2000 N.

After measuring the movement of the physiologic patella a total knee arthroplasy was performed on the specimens (Interax^® ^I.S.A. size 500, Stryker/Howmedica, Limerick, Ireland). The femoral component, which has straight femoral groove and multiple radii in sagittal plane, was implanted with an external rotation of 3 degrees relative to the posterior femoral condyles, the tibial component was implanted at zero degrees to the mechanical axis with no tibial slope of the tibial baseplate. The mobile bearing inlay used is capable of sliding anterior-posterior as well as rotating on the tibial baseplate. A prosthesis was fitted to each specimen in conjunction with a 8 mm PE-inlay, whereby the implantation was performed according to the manufacturers guidelines for opening the knee joint using a mediopatellar incision. The patella was left unresurfaced and relative movement of the center of the patella was measured a second time.

**Figure 3:**
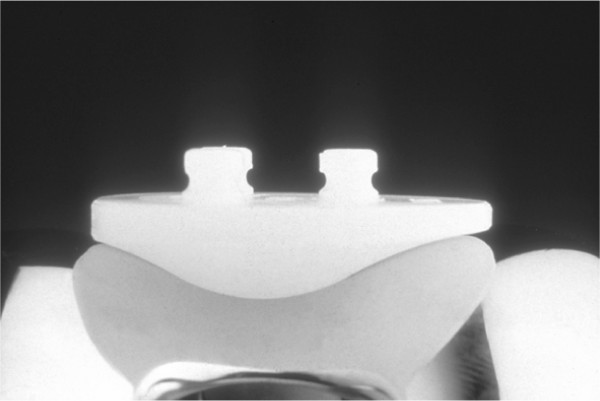
PE patellar inlay of the Interax^®^-Prosthesis system set in the femoral component groove, showing the wide lateral flange

After this test cycle, the patella was resurfaced with the optional included patella inlay. This inlay is a all-polyethylene inlay with a symmetrical shape in the sagittal plane and a 1.5 times wider lateral than medial facet (Fig. 3). The patella surface was resected at a height of 9 mm to allow a size 300 patella-inlay to be fitted and to restore the original thickness of the unresurfaced patella. The inlay was fixed with bone cement with a medial offset of 5 mm relative to the bony center of the patella. Again the relative movement of the center of the patella was measured.

After calculating the path and the relative rotational motion of the center of the patella, means were compared using the nonparametric Wilcoxon test for repeated measures at a significance level of α = 0.05.

## Results

**Figure 4:**
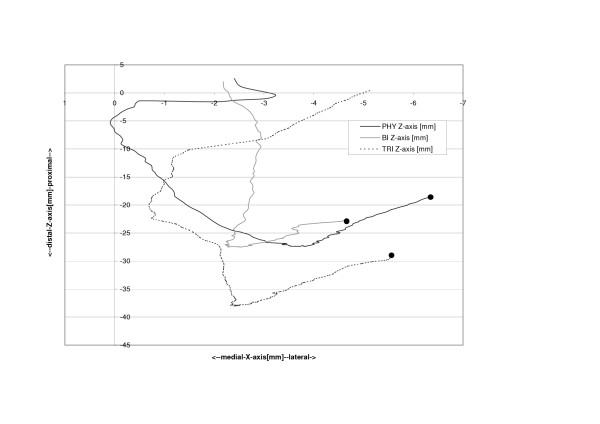
Patella path in coronal plane relative to the starting position (PHY = physiologic patella, BI = unresurfaced patella after TKA, TRI = resurfaced patella after TKA). •(dot) indicates 120° knee flexion, end maximum extension.

**Figure 5:**
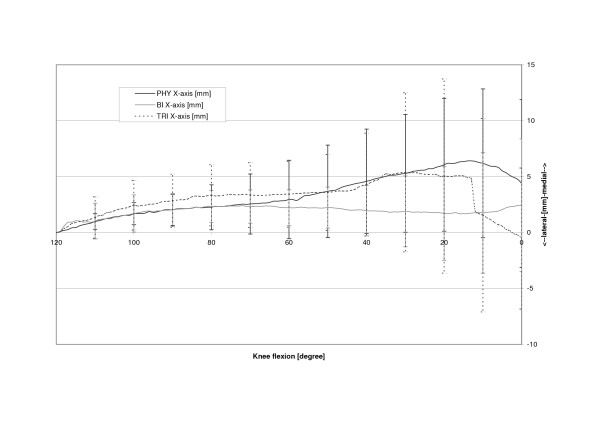
Mediolateral shift of the physiologic (PHY), unresurfaced (BI) and resurfaced patella (TRI) after TKA.

**Figure 6:**
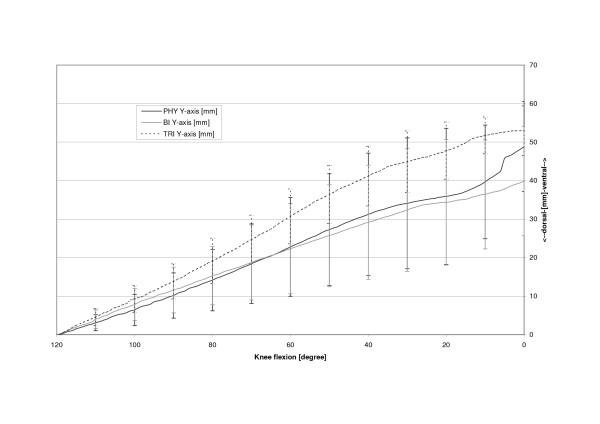
Anteroposterior shift of the physiologic (PHY), unresurfaced (BI) and resurfaced patella (TRI) after TKA.

**Figure 7:**
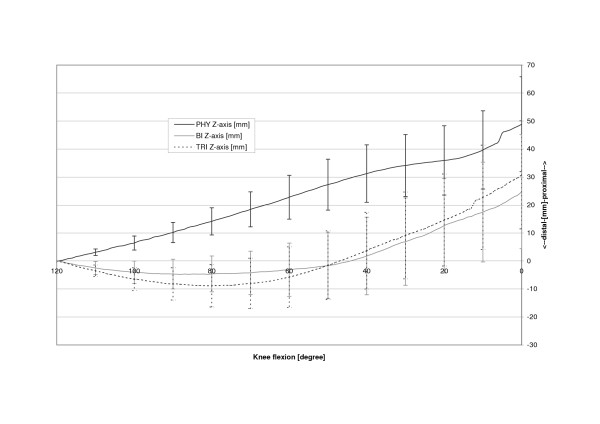
Proximodistal shift of the physiologic (PHY), unresurfaced (BI) and resurfaced patella (TRI) after TKA.

**Figure 8:**
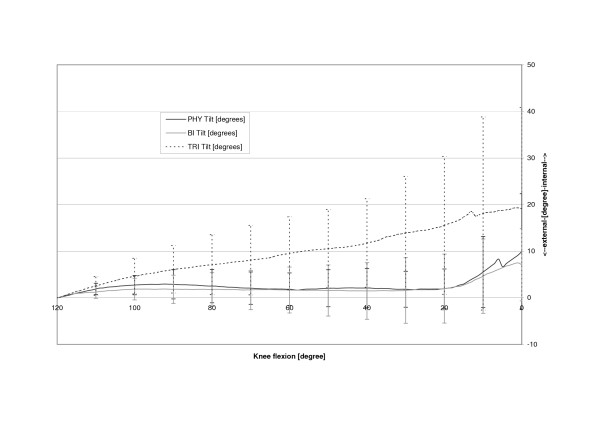
Tilting about the Z axis of the physiologic (PHY), unresurfaced (BI) and resurfaced patella (TRI) after TKA.

**Figure 9:**
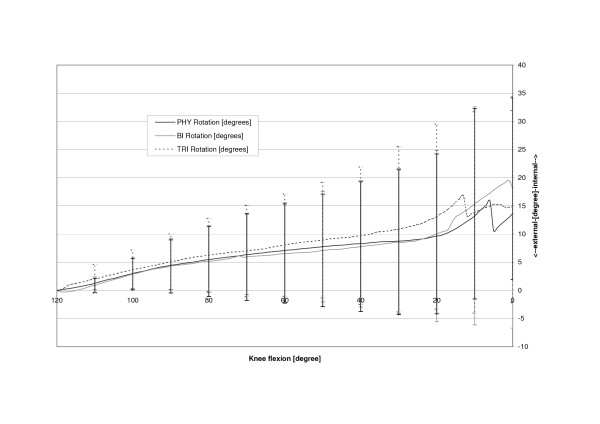
Rotation about the Y axis of the physiologic (PHY), unresurfaced (BI) and resurfaced patella (TRI) after TKA.

**Figure 10:**
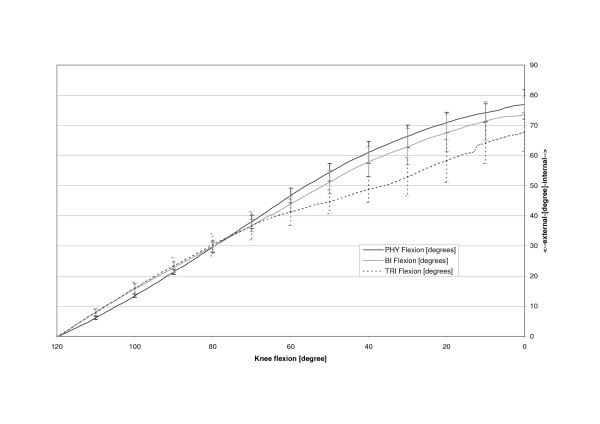
Flexion about the X axis of the physiologic (PHY), unresurfaced (BI) and resurfaced patella (TRI) after TKA.

The physiologic patella (PHY) was observed to move on a semilunar path in the coronal plane (Fig. 4). Relative motions to the femoral centre in the physiologic femoral groove of up to 6.4 mm (SD 6.4 mm) medially were recorded. The most medial point relative to the starting point at 120 degrees of knee flexion was reached at 14.0 degrees flexion after which the patella again moved laterally with further extension (Table 1, Fig. 5). The unresurfaced physiologic patella after TKA (BI) had significantly less (p = 0.04) maximum medial movement of 2.4 mm (2.4 mm, SD 5.9 mm, p = 0.04), with its most medial position being achieved at full extension. The path of this patella was less curved and proceeded almost linearly towards full extension. With a polyethylene resurfacing (TRI) the patella moved on a similar path to the physiologic patella with a maximum medial shift of 5.4 mm (SD 7.1 mm, p = 0.50) being attained. Movements along the Y-axis did not significantly differ with dependence on TKA or patella resurfacing (Fig. 6). Movement along the Z-Axis, which represented the proximodistal direction decreased after TKA. The physiologic patella moved up to 48.9 mm (SD 17.0 mm) proximal while after implantation the unresurfaced patella moved only up to 24.8 mm (SD 20.4 mm, p = 0,33) and the resurfaced patella up to 30.8 mm (SD 19.3 mm, p = 0,47) (Fig. 7). The physiologic patella (PHY) continuously internally tilted up to 10.0° (SD 12.4°) during extension of the knee (Fig. 8). After TKA the patella showed similar (p = 0.34) continuous internal tilting of up to 7.5° (SD 8.7°) until full extension. The resurfaced patella (TRI) showed an internal tilting up to 19.3° (SD 21.7°) during extension (p = 0.50). No significant differences in rotations about the X- and Y-axis of the patella were observed (Fig. 9 and 10).

**Table 1:** Maximum movements and rotations of the physiologic (PHY), unresurfaced (BI) and resurfaced (TRI) patella after TKA.

	X-axis		Y-axis		Z-axis		Tilt		Flexion		Rotation	
	MAX med.	SD	MAX ant.	SD	MAX prox.	SD	MAX int.	SD	MAX	SD	MAX int.	SD
	[mm]	[mm]	[mm]	[mm]	[mm]	[mm]	[degr.]	[degr.]	[degr.]	[degr.]	[degr.]	[degr.]
PHY	6.4	6.4	48.9	11.7	48.9	17.0	10.0	12.4	77.0	4.9	15.9	21.2
BI	2.4	5.9	39.9	14.2	24.8	20.4	7.5	8.7	73.5	5.9	19.5	18.2
TRI	5.4	7.1	53.0	6.3	30.8	19.3	19.3	21.7	67.9	6.5	16.8	17.8

## Discussion

In this study, the path and rotation of the centre of the physiologic patella as well as that of the unresurfaced and resurfaced patella after TKA were measured under dynamic conditions in an *in vitro *knee-extension simulation with physiological quadriceps force. The physiologic patella moved in a semilunar bow shaped path from its starting position to a more medial position, before finally moving back to its original medial-lateral position in the course of the from flexion to full extension knee motion. These results correlated with the findings of Patel et *al. *who showed a similar path of the patella in vivo with a maximum medial shift of 3.2 mm at 30 degrees of flexion [19]. During extension, the patella was observed to tilt medially up to 4.2 degrees.

A limitation of this study of patellar movement is that the simulated extension cycle did not include a weight bearing component, and that the co-contraction of the hamstrings which could also have an additional effect on the patellar path were not considered. Furthermore, the individual contribution of the M. vastus medialis and lateralis were not considered and the resulting quadriceps force vector was directed along the axis of the femur. Nonetheless, unlike other *in vitro *simulations, physiological muscle forces were applied (up to 1500 N), and the kinematics of knee motion attained using this simulator have been shown to be similar to physiological on physiological specimens [20]. In addition, measurement of patellar movement revealed only motions relative to the individual starting points of each patella at 120 degrees of flexion. But in this study as well, motion of the physiologic patella was comparable to the patellar path reported for physiologic patellae of *in vivo *investigations [13]. In the present study, we observed significant changes in medial shift of the patella in the femoral groove after TKA. The unresurfaced patella shifted significantly less medially during extension accompanied by less of a bow shaped path in the femoral groove than compared to the physiologic patella and the resurfaced patella. These differences may be explained by the fact that the femoral groove of the Interax^®^-prosthesis used in this study is symmetrical, in contrast to the physiologic joint. This symmetrical femoral groove functions as a new guideline for the patellar path and represents a simplification of the anatomic shape of physiologic femoral groove which has a higher lateral flange. In the physiologic knee, the patella is guided by this higher lateral flange which pushes the patella in a medial direction with extension. These results contrast to the findings of Omori et *al.*, who showed no significant changes in movement of an unresurfaced patella after TKA with the Genesis^®^-Prosthesis system (Smith&Nephew Richards, Memphis, USA) [17]. The Genesis^®^-Prosthesis system has a more anatomical femoral groove with a higher lateral flange, which may explain why the unresurfaced patella moved more medially (physiologically) in that system.

The results of this study further showed that resurfacing of the patella resulted in a similar path with a medial shift similar to the physiologic knee. As the patella inlay of the Interax^®^-Prosthesis system has a wider lateral facet and an optimized surface fit to the femoral component groove, the patella shifted more medially in a bow shaped path of motion in a similar manner to the physiologic patella. With a resurfaced patella the path of motion observed correlated with the findings of other kinematic studies of patellar path after resurfacing the patella [1,6,12,13,15,17,21,22]. Nonetheless, while translation was similar to physiological, the resurfaced patella tilted two times more internally than the physiologic patella during extension, which was also in contradiction to findings of Omori et *al. *who again found no different tilting after resurfacing [17]. Again, as with the explanation of the different medial shift observed, the asymmetrically shaped inlay in the horizontal plane of the Interax^®^-Prosthesis investigated in this study may explain the larger tilt angles observed relative to those reported for the Genesis^®^-Prosthesis system which has a symmetrical inlay. Because of the asymmetrical shaped patella with a wider lateral flange of this system, the lateral facet could be oversized. As the patella component has a better congruity with the femoral groove than the unresurfaced patella, the patella is better guided, as evidenced by the restoration of mediolateral shift. Concomitantly, larger patellar tilt was seen as the relatively oversized lateral facet was leveled.

Furthermore, a decreased proximal shift of the patella was observed after TKA in the present study, which could potentially lead to a change of the tibiofemoral joint line and produce a patella baja. This may occur as a result of implanting a higher inlay than present in the knee joint before implantation in order to provide stable knee joint conditions, and could lead to increased contact forces on the patellar surface [23-26].

This study demonstrated the ability of a patella resurfacing to restore the physiological mediolateral shift of the patella after TKA. It is hypothesized that the restoration of the physiologic kinematics will result in less patellofemoral complications caused by maltracking of the patella [4]. To date, it is unclear if the higher internal tilt has a negative influence on patellar and knee kinematics, whereas it can be hypothesized that because of lateral oversizing, this internal tilt leads to higher mechanical stresses on the lateral facet of the patellar component [9,12,13]. In addition, the internal tilt could reduce the contact area of the patellar component, which could lead to additional higher contact stresses [17]. Further investigations are planned in order to verify whether this patellar resurfacing design does in fact result in improved *in vivo *kinematics and the clinically anticipated reduction in patellofemoral complications.

## Conclusion

The results of this study suggest that resurfacing of the patella during TKA can result in a restoration of the physiologic mediolateral shift of the patellofemoral joint while angulation of the patella was left in an unphysiological state.

## List of abbreviations

BI unresurfaced patella after total knee arthroplasty

MPa Megapascale

PE Polyethylene

PHY physiologic patella

SD standard deviation

TKA total knee arthroplasty

TRI resurfaced patella after total knee arthroplasty

## Competing interests

The author(s) declare that they have no competing interests.

## Authors' contributions

Dr. Sven Ostermeier performed the test setup, observed the test cycles and made the biomechanic and statistic analysis. He also wrote the primary manuscript.

Olaf Buhrmester performed the test cycles and developed several mechanical devices for the positioning system.

Dr. Christof Hurschler helped with the biomechanical and statistical analysis. He performed the correction of the final manuscript.

Dr. Christina Stukenborg-Colsman performed the test setup and helped in terms of clinical background.
